# The Multiple Roles of Pericytes in Vascular Formation and Microglial Functions in the Brain

**DOI:** 10.3390/life12111835

**Published:** 2022-11-09

**Authors:** Yuki Hattori

**Affiliations:** Department of Anatomy and Cell Biology, Nagoya University Graduate School of Medicine, Nagoya 466-8550, Japan; ha-yuki@med.nagoya-u.ac.jp; Tel.: +81-52-744-2035

**Keywords:** blood–brain barrier, blood vessel, brain development, cortex, endothelial cell, microglia, neurovascular unit, pericyte, vascular structure

## Abstract

In the capillary walls, vascular endothelial cells are covered with mural cells, such as smooth muscle cells and pericytes. Although pericytes had been thought to play simply a structural role, emerging evidence has highlighted their multiple functions in the embryonic, postnatal, and adult brain. As the central nervous system (CNS) develops, the brain’s vascular structure gradually matures into a hierarchical network, which is crucial for the proper development of neural lineage cells by providing oxygen and nutrients. Pericytes play an essential role in vascular formation and regulate blood‒brain barrier (BBB) integrity as a component of the neurovascular unit (NVU), in collaboration with other cells, such as vascular endothelial cells, astrocytes, neurons, and microglia. Microglia, the resident immune cells of the CNS, colonize the brain at embryonic day (E) 9.5 in mice. These cells not only support the development and maturation of neural lineage cells but also help in vascular formation through their extensive migration. Recent studies have demonstrated that pericytes directly contact microglia in the CNS, and their interactions have a profound effect on physiological and pathological aspects. This review summarizes the function of pericytes, focusing on the interplay between pericytes and microglia.

## 1. Introduction

As the central nervous system (CNS) develops, the brain’s vascular system gradually matures into a hierarchical network, which is crucial for the proper development of neural lineage cells by providing oxygen and nutrients. The formation of blood islands, which is known as vasculogenesis, is initially observed in the extraembryonic yolk sac around embryonic day (E) 6.5 in mice [1]. Vasculogenesis includes mesoderm formation, the differentiation of mesoderm-derived angioblasts into endothelial cells (ECs), and their organization into vascular tubes [2,3]. In the embryonic brain, similar to other embryonic tissues, once angioblasts differentiate into ECs, they enter the perineural epithelium of the embryonic brain [4,5]. Next, vascular ECs form a primary perineural vascular plexus that encompasses the neural tube, and are dispersed in the whole brain structure on E7.5–E8.5 in mice [6]. Vascular elongation, branching, and remodeling start at E9.5 and continue to the postnatal stage [1,7]. Subsequently, mural cells, such as pericytes and smooth muscle cells (SMCs), are recruited to the vascular endothelium, and they encompass the vascular tube [5]. Such complicated processes finally lead to the establishment of a functional vascular system.

Pericytes are the cells that cover vascular ECs throughout the body, including the brain [8]. These cells are different from vascular ECs and SMCs. Pericytes are multifunctional cells that play essential roles in vascular formation and the maintenance of blood‒brain barrier (BBB) integrity.

Microglia are resident immune cells in the CNS. Microglia originate from erythromyeloid progenitors (EMPs) in the yolk sac in the early embryonic stage, and these progenitors then colonize the CNS through their extensive migration and proliferation during development. Recent studies have demonstrated that pericytes directly interact with microglia in the CNS. This review describes the function of pericytes, especially focusing on the interplay between pericytes and microglia.

## 2. What Is a Pericyte?

**Figure 1:**
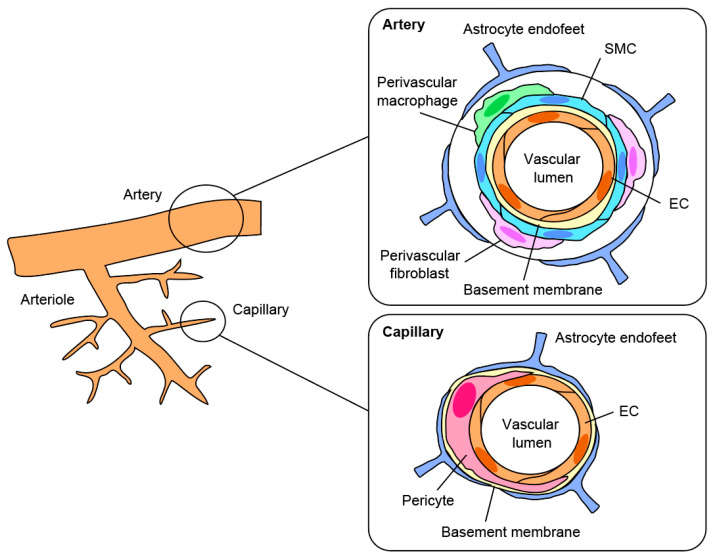
The BBB structure in the brain. Schematic of the BBB structure. The structures in the artery (**right upper**) and capillary (**right bottom**) are shown, respectively. Pericytes are distributed in capillaries.

Pericytes were first identified by Charles-Marie Benjamin Rouget in the 1870s. These cells were described as rouget cells [9]. In the 1920s, they were later called pericytes by Zimmermann, who stated that he defined various cellular morphologies as pericytes [8]. Pericytes are mural cells that surround the vascular ECs in capillaries and directly communicate with ECs through tight connections [10,11] (Figure 1). In collaboration with vascular ECs, neural lineage cells, and the extracellular matrix, pericytes form the neurovascular unit (NVU) to maintain a highly selective BBB and establish cerebral homeostasis. Recent studies have revealed that pericytes not only function in a structural role but also play a critical role at various points. For example, pericytes contribute to the rigorous integrity of the BBB, which limits the invasion of harmful substances and provides a suitable ionic environment that supports neuronal activity, regulates vascular formation and cerebral blood flow (CBF), and facilitates neuronal inflammation. In addition, a growing body of evidence indicates that pericytes directly interact with neural lineage cells and microglia.

Pericytes have been studied for almost 150 years, but their ambiguous identification makes it difficult to investigate their characteristics and function. Pericytes express several specific markers, such as chondroitin sulfate proteoglycan (neuron-glial antigen 2; NG2), platelet growth factor receptor beta (PDGFRβ), Desmin, RGS5, CD13, CD146, and RGS5 [12], but do not have unique molecular markers. Some of these markers are also expressed in other cells, such as ECs and SMCs. However, the detection of multiple markers is useful. For example, pericytes can be identified by the expression of two cell surface proteins: PDGFRβ and NG2. Neither marker is specific to pericytes: PDGFRβ is expressed by fibroblasts, whereas NG2 is expressed by oligodendrocyte progenitor cells (OPCs). Both of them are expressed at lower levels by vascular SMCs [13]. However, the combination of other markers that are specific for oligodendrocyte progenitors and SMCs helps us to identify pericytes. SMCs also express alpha smooth muscle actin (α-SMA), CD13, CD146, and Desmin, whereas OPCs express platelet growth factor receptor alpha (PDGFRα). A very recent paper showed that mural cells are represented in both glucagon-like peptide-1 receptor (Glp1r)^+^ and glucose-dependent insulinotropic polypeptide receptor (Gipr)^+^ populations. Glp1r^+^ mural cells are largely SMCs, while the majority of Gipr^+^ mural cells are pericytes, indicating that Gipr may be a useful marker of pericytes [14].

Little is known about the exact embryonic origin of pericytes, but several studies using lineage tracing methods have suggested distinct developmental sources. During ontogenesis, pericyte subtypes mainly belong to two embryonic germ layers: neural crest cells and the mesoderm. Quail chick chimeras and lineage tracing studies demonstrated that pericyte origins differ among pericytes that are localized in a region: pericytes positioned in the forebrain arise from neural crest cells, while those in the brainstem, spinal cord, and mid-brain are derived from the mesoderm [15,16,17]. Recent single-cell RNA sequencing studies revealed the heterogeneity and multitasking ability of pericytes [18,19,20,21,22]. A previous study based on cell linage tracing analysis using several transgenic mice revealed that pericytes have more heterogenous characteristics than previously thought [23]. Another work demonstrated that pericytes exhibited heterogeneous properties within the same tissue [24]. Thus, more detailed analyses of the relationship between pericyte origin and heterogenic function may help to better understand pericytes’ characteristics.

## 3. The Functions of Pericytes in Vascular Formation

Pericytes contribute to vascular stability, angiogenesis, and the maintenance of cerebral microcirculation in the physiological and pathological conditions. This section describes their functional roles in blood vessel formation.

### 3.1. Vascular Development

**Figure 2:**
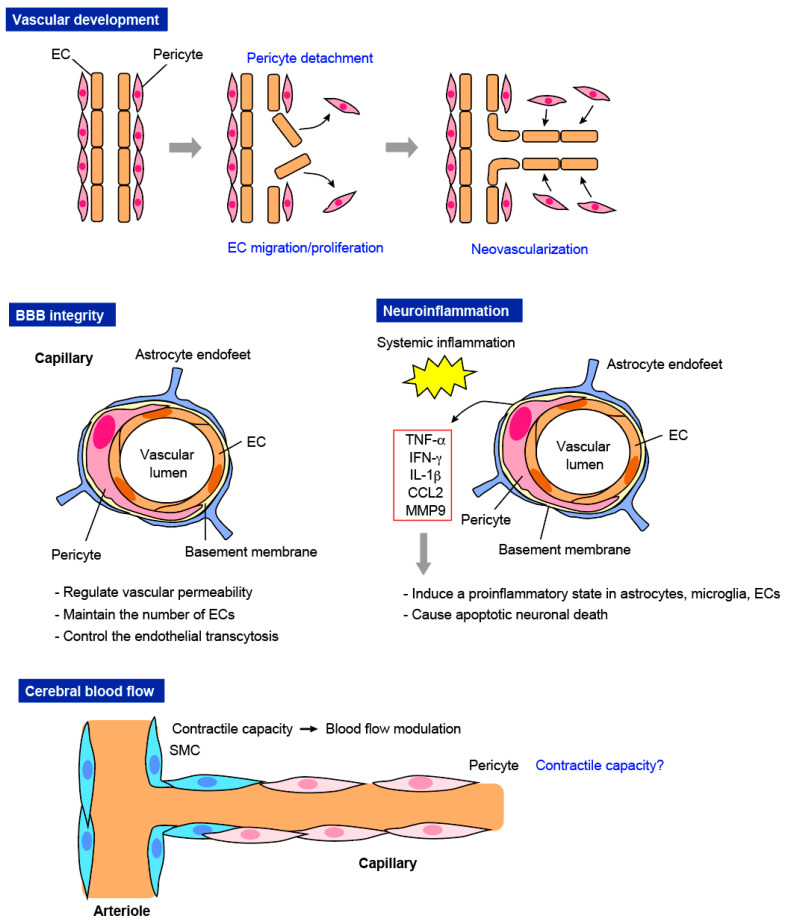
The functions of pericytes on blood vessels. Pictures summarize the suggested functions of pericytes on blood vessels. Pericytes play a key role in vascular development, blood–brain barrier (BBB) integrity, and neuroinflammation. Their contribution to cerebral blood flow (CBF) is a controversial issue so far.

First, pericytes play an essential role in the generation of nascent blood vessels (Figure 2). Pericytes first detach from the ECs that form the blood vessel wall. Their detachment triggers the migration of vascular ECs toward the surrounding matrix to generate new blood vessels [25,26]. Following this, pericytes are again recruited to ECs and then remodel the vascular tubes and stabilize the structure [27,28,29]. A recent study demonstrated that the single-cell depletion of pericytes by laser ablation caused pericytes to extend their processes toward the pericyte-uncovered regions surrounding ECs to stabilize the vascular structure and vessel diameter [30].

ECs release growth factors to attract pericytes to newly formed blood vessels in order to stabilize the vascular structure [10,29]. Previous studies have demonstrated that the PDGFB/PDGFRβ axis plays an essential role in pericyte recruitment. Mice that lacked PDGF-B or PDGFRβ showed severe deficits in the pericyte coverage of blood vessels, endothelial hyperplasia, and abnormal vascular morphogenesis, including microaneurysm, which led to widespread microvascular leakage and edema [25,27,31,32]. Mice with null and hypomorphic alleles of *Pdgfrb*, which had defects in pericyte generation, showed that pericytes were required for BBB formation and that pericyte coverage with the blood vessels determined vascular permeability [33]. Transforming growth factor-β (TGF-β) induces the differentiation of mesenchymal cells toward an SMC/pericyte lineage. A recent study tested the hypothesis that TGF-β not only induced SMC differentiation but also stabilized capillary-like structures in a three-dimensional (3D) model of in vitro angiogenesis [34]. Another study showed that fluorescence-activated cell sorting (FACS)-isolated myeloid cells and their progenitors from embryonic skin differentiated into pericytes through TGF-β signaling in culture [23]. The researchers demonstrated that type 2 TGF-β receptor (TGFBR2) mutants exhibited deficient pericyte development in the skin vasculature. Furthermore, another study demonstrated that extracellular vesicles released from ECs, driven by inflammation, affected the cellular status of pericytes [35].

On the other hand, pericytes also regulate EC proliferation, survival, and differentiation. Pericytes promote angiogenesis by releasing vascular endothelial growth factor (VEGF) and neurogenic locus notch homolog protein (NOTCH) 3 in the adult brain [36,37]. These cells also induce the sprouting and stabilization of ECs by secreting VEGF, TGFβ, and angiopoietin 1 (ANGPT1) [38,39,40,41,42,43]. Another study showed that silencing Tie2 in pericytes, which express the functional Tie2 receptor, resulted in a promigratory phenotype of ECs in human and murine models, indicating that Tie2 expression on pericytes controls sprouting angiogenesis, as determined by in vitro sprouting and in vivo spheroid assays [43]. Pericytes also control the cell cycle of ECs [39]. These factors affect each other’s mitotic rates, differentiation, and growth arrest. Pericytes have also been suggested to suppress EC growth. In cocultures, pericytes inhibited all EC proliferation. These results suggest that pericytes can modulate EC growth via a mechanism that requires contact or proximity.

Overall, the bidirectional interaction of vascular ECs and pericytes is substantially required for vascular formation and stabilization.

### 3.2. BBB Integrity

The tight and adherens junctions forming the BBB tightly control the influx and efflux of biological substances, such as ions and molecules, and the infiltration of circulating cells between the bloodstream and the brain parenchyma, thereby providing a favorable environment for brain cells [44,45]. This rigorous integrity of the BBB is controlled by the NVU, a collection of various cell types, such as ECs, pericytes, astrocytes, neurons, microglia, and perivascular macrophages [46,47,48,49].

In the physiological state, pericytes contribute to BBB integrity. First, pericytes regulate tight junction integrity and/or maintain EC alignment. Pericytes adjust the number of ECs and modulate astrocyte endfeet positioning through their release of signaling factors [50]. Pericyte loss causes the failure of the formation of tight junctions between ECs, leading to an abnormal increase in BBB permeability [51]. Pericyte damage leads to late endothelial changes and ultimately choriocapillaris loss [52]. Pericyte depletion using a double promoter strategy for PDGFRβ and NG2 led to acute blood–brain barrier breakdown, which was associated with rapid neuronal loss due to the decrease in pericyte-derived pleiotrophin, a neurotrophic growth factor [53].

Second, pericytes regulate the endothelial transcytosis of vesicles and contribute to the clearance of toxic species from the brain [54]. Adult pericyte-deficient mice demonstrated that pericyte loss caused BBB breakdown, associated with the accumulation of serum proteins, several vasculotoxic molecules, and neurotoxic substances, which led to secondary neurodegenerative changes [36]. Third, pericytes directly regulate the infiltration of immune cells. Pericytes inhibit the expression of molecules that cause an increase in immune cell infiltration [33].

In the pathological context, pericytes impact BBB integrity through their activation. First, pericytes produce inflammatory molecules, which leads to BBB breakdown. Pericytes exposed to hyperglycemia and advanced glycation and products (AGEs) displayed diminished expression of integrin a1, PDGFRβ, and connexin 43, leading to BBB injury [55,56]. BBB compromise was demonstrated in ex vivo, in vitro, and in vivo diabetes mellitus models, and the combination of human immunodeficiency virus type 1 (HIV1) and diabetes enhanced BBB injury via effects on the brain endothelium and pericytes. Second, pericytes replenish the cells in the NVU to maintain the environment through their stem cell-like properties and activities [20]. Under ischemic/hypoxic conditions, pericytes can acquire multipotential stem cell activity and differentiate into major components of the NVU in the BBB [57,58]. Thus, pericytes contribute to BBB integrity by regulating permeability and display a complementary role/replenishment in both physiological and pathological conditions.

### 3.3. Neuroinflammation

Neuroinflammation is primarily instigated by the abnormal activation of microglia, astrocytes, and infiltrating leukocytes [59,60]. In collaboration with these cells, pericytes produce several mediators that trigger leukocyte infiltration [61]. In adult pericyte-deficient *Pdgfb*^ret/ret^ mice, pericytes limited leukocyte extravasation and/or transmigration into the CNS, not only in the physiological state but also in a pathological context [62]. In *Pdgfrb*-Cre:*Ccl2*^fl/fl^ mice, an increase in excitatory synaptic transmission caused by LPS administration was markedly diminished, suggesting that PDGFRβ^+^ cells sense the foreign invasion and transmit the inflammation to the CNS by secreting CCL2 (chemokine CC chemokine ligand 2, MCP1) [63]. A study using a 3D model of the human BBB demonstrated that pericytes can enhance the secretion of granulocyte colony stimulating factor (G-CSF) and interleukin-6 (IL-6) when co-cultured with endothelium [64]. Other studies have demonstrated that the immunomodulatory factors secreted by pericytes, including IL-1β, TNF-α, interferon-γ (IFN-γ), and IL-6, induce a proinflammatory state in astrocytes, microglia, and ECs, and cause apoptotic neuronal death [65,66]. A recent study showed that chondroitin sulfate proteoglycans (CSPGs), which are extracellular matrix proteins enriched within inflamed perivascular cuffs, increased the levels of proinflammatory chemokines/cytokines in pericytes in culture. Furthermore, pericytes stimulated with CSPGs enhanced macrophage migration [67]. The brain’s immune-privileged status is compromised when pericytes are lost or lymphatic vessels are dysregulated [68].

### 3.4. CBF Modulation

Rouget regarded pericytes as contractile cells [9]. Although SMCs that are positioned at arterioles were traditionally thought to regulate CBF [69], it is a controversial issue so far whether pericytes also regulate vessel diameter and CBF via their contractility or not.

Previous studies have reported that pericytes can flexibly contract and relax to modulate CBF in response to surrounding environmental changes [46,70,71,72,73]. Pericytes, when they react to neurotransmitters, dilate capillaries and increase the local amount of CBF [74]. Another study demonstrated that pericytes have an impact on CBF by altering the stiffness of the capillary wall, thereby changing the capillary transit time [75]. In *Pdgfrb*-deficient mice, pericyte loss reduced the CBF, with rapid neurovascular uncoupling [36,76].

Using two-photon microscopy, pericyte stimulation induced increases in synaptic activity and capillary dilation, supporting an active role of pericytes in cerebrovascular control [77]. A previous study using inducible pericyte-deficient mice obtained from a pericyte-specific PDGFRβ-Cre mouse line crossed with Cre-inducible diphtheria toxin receptor mice (iDTR mice) demonstrated the functional role of pericytes in CBF regulation, suggesting that pericyte loss may lead to stroke, Alzheimer’s disease (AD), and other neuronal disorders [72]. Furthermore, another study reported that pericytes act as regulators of CSF at the border between arterioles and capillaries by controlling the length and width of the enclosed vessel segment [78].

In contrast, several recent papers raised the possibility that pericytes do not have contractile properties. Although pericytes have been reported to express contractile proteins, including α-SMA, tropomyosin, and myosin [79,80], difficulty in discrimination between pericytes and SMCs may lead to different interpretations. Fernández-Klett et al. reported that SMCs but not pericytes were responsible for the CBF increase [81]. Another report also supported this idea, and proposed that arteriolar SMCs mainly control CBF [82]. Thus, the establishment of new techniques to enable us to separately evaluate pericytes’ functions from other cells is required.

## 4. The Interplay between Microglia and Pericytes

### 4.1. Microglia to Pericytes

Microglial cell bodies are positioned along small blood vessels in the healthy mouse brain. The number of papers that have investigated the interaction between microglia and pericytes is still relatively low, but recent evidence has demonstrated the importance of the association of pericytes with microglia [83]. This section summarizes the papers that have investigated how microglia contribute to or affect pericyte function.

First, microglia are known to induce pericyte apoptosis. IL-1β, which is produced by ECs and microglia, induced pericyte apoptosis via nuclear factor-kappa B (NF-kB) activation under high-glucose conditions, thereby increasing endothelial permeability in diabetic retinopathy [84]. TNF-α is a potent inducer of pericyte apoptosis in diabetic retinas based on gene expression analysis, and signal transducer and activator of transcription 3 (STAT3) activation in microglia increases TNF-α expression in diabetic retinas. TNF-α released from microglia induced pericyte apoptosis by downregulating AKT/p70S6 kinase signaling. Using microglia-specific STAT3-deficient mice, the researchers showed that STAT3 ablation in microglia downregulated TNF-α expression and reduced pericyte apoptosis in diabetic retinas, indicating that STAT3 activation in microglia was essential for pericyte apoptosis in diabetic retinas through increased TNF-α expression [85].

Second, microglia modulate pericyte maturation and change their phenotype. The presence of maturation transition and stemness features in pericytes could maintain blood‒brain barrier functionality during different pathologies. In vitro culture of rat pericytes with conditioned media of microglia that were induced to an anti-inflammatory state promoted pericyte maturity [86]. Another study showed that APOE4 derived from microglia not only disturbed lipid homeostasis in macrophages and SMCs, leading to exacerbated systemic inflammation and atherosclerotic plaque formation, but also contributed to pericyte activation, disturbing BBB integrity [87].

Third, microglia regulate the number of pericytes. Administration of liposomal clodronate over 5 weeks increased the number of activated CD74^+^ microglia and subsequently led to pericyte loss on the capillaries. Activated microglia induced the pleiotropic protective pathways that support vasoprotection [88].

Fourth, microglia disturb pericyte-mediated microcirculation. Microglia release TNF-α after traumatic brain injury, which promotes neuroinflammation and oxidative stress by activating downstream inducible nitric oxide (iNOS)/NF-kB signaling, leading to pericyte-mediated disturbances in cerebral microcirculation [89].

Thus, microglia can regulate pericyte function in physiological and pathological states.

### 4.2. Pericytes to Microglia

In contrast, pericytes reciprocally regulate microglial function. This section summarizes recent evidence showing the contribution of pericytes to microglial development and function.

#### 4.2.1. Pericytes as Sensors of Systemic Inflammation

**Figure 3:**
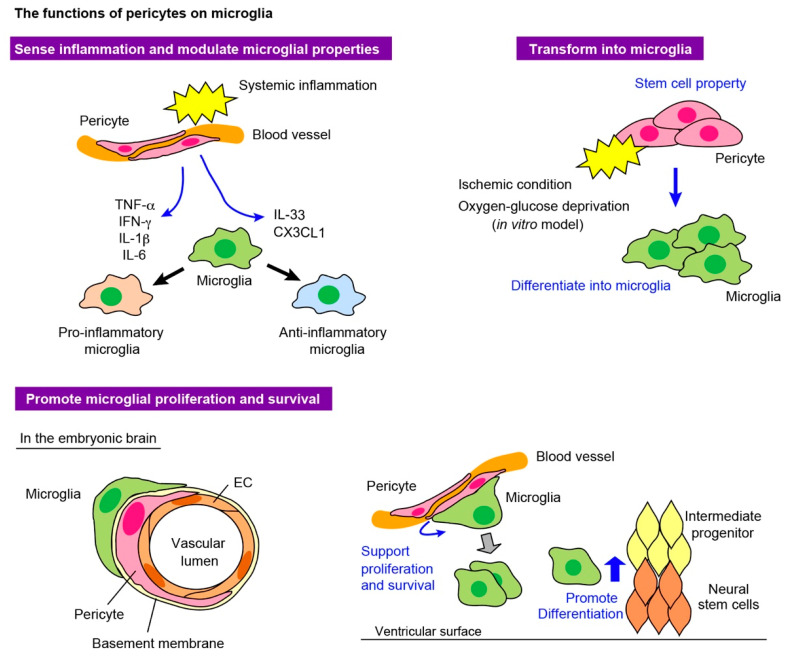
The interactions between microglia and pericytes. The schematic summarizes the interaction between microglia and pericytes in the physiological and pathological states.

Pericytes release inflammatory mediators in response to systemic inflammation, and alter the characteristics of surrounding cells, including microglia [63,90,91] (Figure 3). In the mouse brain, pericytes alter the motility of microglia that are positioned along blood vessels by secreting CCL2, which triggers microglial activation in infection [63,92]. Moreover, pericytes release other cytokines, such as TNF-α, IFN-γ, IL-1β, and IL-6, to induce an inflammatory state in the surrounding cells, including microglia, and cause neuronal apoptosis [65,66]. Pericytes also secrete anti-inflammatory molecules and induce microglia to acquire anti-inflammatory properties, and microglia produce interleukin 33 (IL-33) and C-X3-C motif ligand 1 (CX3CL1) in a mouse model [90,93,94,95]. Furthermore, the depletion of pericytes induced inflammatory responses in ECs and the perivascular infiltration of macrophages in mouse retinal vessels, suggesting that pericytes exert anti-inflammatory effects on these cell types in the physiological state [96]. Clinical evidence has revealed the accumulation of pericytes with altered morphology in the cerebral vasculature of patients with interactable epilepsy [97,98]. Experimental studies using an epilepsy animal model showed that cerebrovascular pericytes changed their phenotypes in response to proinflammatory cytokines such as IL-1β, TNF-α, and IL-6, and underwent redistribution and remodeling.

#### 4.2.2. Pericytes Acquire Microglial Properties

Through in vitro and in vivo studies, pericytes have been shown to differentiate into multiple cell types, including angioblasts, neural progenitors, vascular cells, microglia, and other glial cells, in response to stimuli and environmental changes [20,57,99,100,101].

Baron et al. initially raised the possibility that pericytes had high plasticity and could transform into microglia via the astrocytic activation process [102]. Previous studies have reported that pericytes, in response to external stimuli, can convert into mesenchymal stem cells, which have the potential to differentiate into various cell types: neurons, astrocytes, microglia, and fibroblast progenitors [20,57]. Both mouse pericytes isolated from ischemic brains and human pericytes cultured under oxygen/glucose deprivation developed stem cell properties through reprogramming. These pericytes have their original mesenchymal properties and multipotential ability to differentiate into both a neural and vascular lineage [57]. Other studies reported that pericytes under ischemic conditions in vitro and in vivo acquired a microglial phenotype with increased phagocytic properties, indicating that pericytes immediately react to environmental changes and contribute to tissue remodeling [58,99]. However, these findings are exclusively based on experiments performed on cells cultured in vitro or on observations made in animals that received transplants of pericytes that were previously propagated in vitro. A very recent study demonstrated that α-SMA^low/undetectable^ pericytes, but not vascular SMCs or fibroblasts, differentiated into microglia-like and macrophage-like cells after stroke [103]. However, there have been no reports showing that pericytes possess multipotency to differentiate into tissue-resident cells in vivo.

However, the cell fate and plasticity of pericytes in vivo is a controversial issue. A previous study reported that FACS-isolated cells expressing Tbx18, a transcription factor that is selectively expressed in pericytes and vascular SMCs in multiple organs of adult mice, behaved similarly to mesenchymal stem cells in vitro, but lineage tracing experiments using an inducible Tbx18-CreERT2 line revealed that pericytes and vascular SMCs maintained their identity with age and in diverse pathological settings, suggesting that these cell types did not significantly contribute to other cell lineages [104]. This study has challenged the current concept of pericyte multipotency and suggests that the plasticity observed in vitro or following transplantation in vivo arises from artificial cell manipulation ex vivo.

As mentioned in the previous section, emerging evidence has demonstrated that pericytes are a heterogeneous population. Accordingly, their origin, diverse functions, and/or various cellular subtypes should be taken into consideration to better understand the complexity of pericytes’ biology.

#### 4.2.3. Pericytes Support Microglial Survival and Proliferation

In the embryonic brain, immunohistochemistry showed that around half of the microglia were attached to capillaries. The proportion of the pericyte coverage area of the capillaries in the E14 mouse cerebral wall was approximately 80% [105]. Importantly, magnified observations revealed that microglia tend to selectively associate with the regions covered with pericytes, raising the possibility that pericytes facilitate microglial development and/or distribution. Indeed, the in vivo pericyte depletion model and in vitro coculture experiment suggested that pericytes promoted microglial survival and proliferation in the brain parenchyma at the embryonic stage.

The blockade of PDGFRβ signaling by the intraventricular injection of anti-PDGFRβ neutralizing antibodies induced pericyte apoptosis. Pericyte loss led to a decrease in microglial density in the cerebral wall. An in vitro cell culture experiment of microglia and pericytes, which were isolated as NG2^+^PDGFRα^−^ cells, demonstrated that the density of microglia was increased in microglia cultured together with pericytes as compared to microglia cultured alone, suggesting that pericytes promote microglial proliferation.

Moreover, pericytes support microglial homeostasis and facilitate microglial function by promoting the differentiation of neural stem cells into intermediate progenitors. In the pericyte-depleted cerebral wall, the numbers of Sox2^+^ neural stem cells and Tbr2^+^ intermediate progenitors were increased and decreased, respectively, suggesting that pericytes indirectly support microglia to efficiently promote the differentiation of neural progenitors.

## 5. Conclusions

This review summarized current research on pericyte function, such as their contributions to vascular formation, BBB integrity, neuroinflammation, and CBF. In the latter part, recent studies on the interplay between pericytes and microglia were described. Pericytes play profound roles not only in blood vessel conformation and BBB integrity but also in proper microglial function and homeostasis in the brain. Since pericytes do not have specific markers that are unique to them, it is difficult to identify and distinguish pericytes from surrounding cells, such as ECs, SMCs, and OPCs. This problem makes it difficult to investigate the characteristics and functions of pericytes. However, recent advancements in techniques and the establishment of transgenic mice have enabled us to more strictly distinguish pericytes from other cells, such as through characterizations using double-specific markers. These newly emerging techniques may become strong tools for pericyte studies. Although there is a controversial issue regarding pericytes’ functions, especially their contribution to CBF or their contractile capacity, in vivo live imaging and/or cell isolation to analyze gene and protein expression using these tools may allow us to investigate whether pericytes indeed have these functions. In addition, recent studies using single-cell RNA sequencing have shown that pericytes have heterogeneity. Lineage tracing experiments demonstrated that pericytes have different origins, such as the mesoderm and neural crest cells. Thus, more detailed analyses of the relationship between pericyte origin and heterogenic function may support a better understanding of pericytes’ characteristics and their specific interactions with other cells. The identification of pericyte dysfunction in pathological and dysfunctional states will offer new perspectives on cell subtype-specific therapeutic approaches.

## Data Availability

Not applicable.
